# Presence of susceptible wild strains of *Anopheles gambiae* in a large industrial palm farm located in Aboisso, South-Eastern of Côte d’Ivoire

**DOI:** 10.1186/s12936-017-1804-1

**Published:** 2017-04-20

**Authors:** Cécile M. A. Sadia-Kacou, Ludovic P. Ahoua Alou, Ako V. C. Edi, Celine M. Yobo, Maurice A. Adja, Allassane F. Ouattara, David Malone, Alphonsine A. Koffi, Yao Tano, Benjamin G. Koudou

**Affiliations:** 1 Laboratoire de Zoologie et Biologie Animale, UFR Biosciences, Université Félix Houphouët Boigny, 22 BP 770, Abidjan, Côte d’Ivoire; 2 Institut Pierre Richet (IPR)/Institut National de Santé Publique (INSP), 01 BP 1500, Bouaké, Côte d’Ivoire; 3 Laboratoire de biologie et cytologie animale, Université Nangui Abrogoua, 02 BP 801, Abidjan 08, Côte d’Ivoire; 4 Centre Suisse de Recherches Scientifiques, Adiopodoumé Km17, 01 BP 1303, Abidjan 01, Côte d’Ivoire; 5 Innovative Vector Control Consortium, Liverpool School of Tropical Medicine, Pembroke Place, Liverpool, L3 5QA UK; 6 Université Nangui Abrogoua, 02 BP 801, Abidjan 08, Côte d’Ivoire

## Abstract

**Background:**

The effectiveness of malaria control programmes through implementation of vector control activities is challenged by the emergence of insecticide resistance. In the South-Eastern region of Côte d’Ivoire, where palm oil plantations remain the predominant agricultural crop, the susceptibility of wild *Anopheles gambiae* sensu lato species is still unknown and thus requires a particular attention. The current study was carried out to address the gap by in-depth characterization of susceptibility level of *An. gambiae* mosquitoes from Ehania-V1 to WHO-recommended doses of six insecticides belonging to available classes and also to screen a subset for target site mutations and possible inhibition of P450 enzymes.

**Results:**

Overall results showed variable resistance profile across WHO-recommended insecticides tested. Mortalities ranged from 8.3% (the lowest mortality was recorded with DDT) to 98% (the highest mortality was recorded with fenitrothion). Importantly, mortality to deltamethrin, an important pyrethroid used in public health for impregnation of mosquito nets was close to 98%, precluding a possible susceptibility to this insecticide, albeit further investigations are required. Pre-exposure of *An. gambiae* s.l. to PBO did not show any significant variation across insecticides (p = 0.002), although a partial increase was detected for alphacypermethrin and bendiocarb, suggesting a low of activity of cytochrome P450 enzymes (p = 0.277). High frequency of *kdr* L1014F was recorded in both *Anopheles coluzzii* (91%) and in *An. gambiae* (96%), associated with *ace*-*1* ^*R*^ G119S mutation at low frequency (<20%).

**Conclusion:**

The high mortality rate to deltamethrin, organophosphate and the non-detection of P450 activity in resistance observed in Ehania-V1 appears as a positive outcome for further control strategies as metabolic-based P450 resistance remains major challenge to manage. These results should help the National Malaria Control Programme when designing strategies for vector control in palm oil areas of Côte d’Ivoire.

**Electronic supplementary material:**

The online version of this article (doi:10.1186/s12936-017-1804-1) contains supplementary material, which is available to authorized users.

## Background

Malaria is a major public health problem in Africa with a burden estimated to over 190 million cases and 395,000 deaths, mainly among children under five [1]. The dominant *Anopheline* mosquitoes responsible for the transmission of malaria parasites in Africa are mainly *Anopheles funestus* and the three complex sibling species *Anopheles gambiae* (formerly *An. gambiae* s.s. S form), *Anopheles coluzzii* (formerly *An. gambiae* s.s. M form), *Anopheles arabiensis*. Vector control based on the use of chemical insecticides through long-lasting insecticide-treated nets (LLIN) and indoor residual spraying (IRS) is one of the most effective measures to prevent malaria transmission [1]. Actually, pyrethroids are the only insecticide class approved for LLINs and also used in IRS programme worldwide [2]. Several studies have demonstrated LLIN and IRS effectiveness in *Anopheles* mosquito control [3, 4], therefore, a reduction of malaria cases and childhood mortality [5]. Strong evidence of impact of current vector control interventions in malaria reduction has recently been reported [6], in which both LLINs and IRS accounted for 78% reduction of malaria cases between 2000 and 2015, and artemisinin-based combination therapy (ACT) accounted for 22%. However, such effort could be compromised if new compounds development is not rapidly undertaken to support the current existing active ingredients. It is also important to note that pyrethroid resistance nearly ubiquitous in various regions was first detected in Cote d’Ivoire, especially in Bouaké town in 1993 [7]. Soon after, several cases of resistance have also been detected in many African countries including Benin [8], Mali [9] and Cameroon [10]. Unfortunately, resistance is also being rapidly reported to other classes of insecticides [11–15].

Up-to-date, key identified resistance mechanisms included (i) mediated amino-acid changes at the target sites of insecticides, leucine to serine (L1014S) or leucine to phenylalanine (L1014F) in the voltage sodium channel gate (*kdr* mutations for pyrethroids and DDT) and glycine to serine at codon 119 (G119S *Ace 1* ^*R*^ mutation for organophosphates and carbamates) and (ii) insecticides detoxification by metabolic enzymes i.e. non-specific esterases, (NSE), mixed-function oxidase (MFO) and glutathione S-transferases (GST). In Côte d’Ivoire, resistance mechanisms have been investigated at both phenotypic [15–17] and genetic level [18].

The sources of selection pressure for resistance remain always a debate. However, the use of insecticides in agriculture has been incriminated [19]. Extensive use of insecticides against agricultural pests, mainly on cotton and irrigated rice farms, has led to the development of resistance in *Anopheles* which negatively impacted malaria control interventions [19].

In the context of a lack of new insecticide classes for malaria control for 30 years and none for the next 6–9 years, vector control relies on improved management of existing insecticides. A key to this is to carry out an in-depth characterization of existing resistance mechanisms. Given the place and role of palm oil production in the economy of Côte d’Ivoire and efforts made to control pests by applying insecticides, it appears crucial to undertake a study to deeply monitor insecticide resistance level in oil palm plantations in Cote d’Ivoire.

This is the first study implemented in an industrial palm oil farm, in order to assess the susceptibility level of the main malaria vectors *An. gambiae* s.l. to World Health Organization (WHO)-recommended insecticide classes, and beyond, the corresponding target-sites mutation frequency.

## Methods

### Study site

**Fig. 1:**
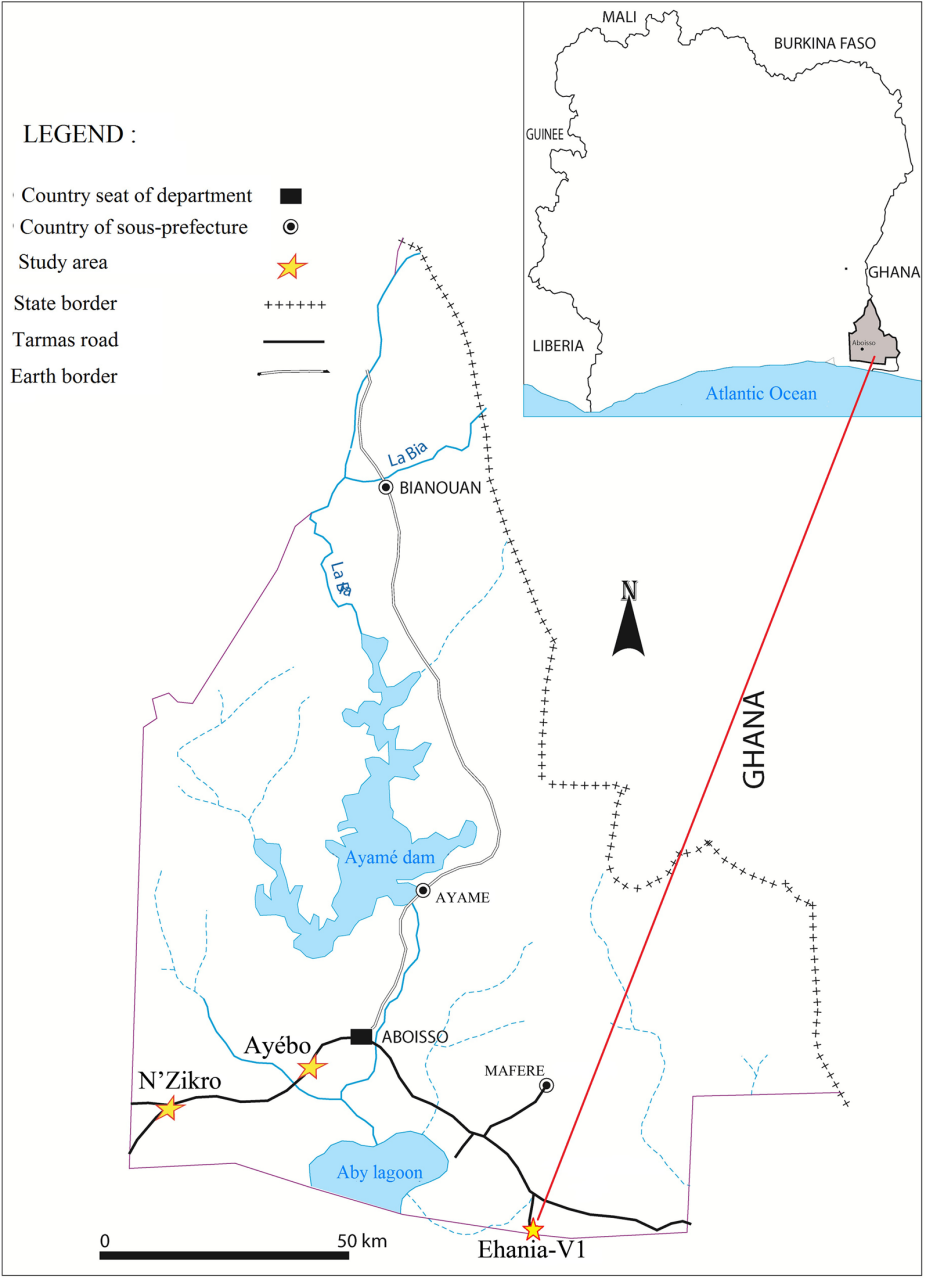
Mapping of Côte d’Ivoire showing Ehania-V1, the study site

Ehania-V1 is located in the South-eastern Côte d’Ivoire, 140 km from Abidjan (Geographical coordinates: longitude: 5°14′N, latitude: 2°46′W), and 60 km from Ghana (Fig. 1). Bordered by Noé and Aboisso, Ehania-V1 is the biggest industrial palm oil farm (30,000 hectares) belonging to the company PALMCI created in 1969 and privatized in 1997. The yield constitutes 32% of the annual palm oil yield of the country. The company plays a role of guidance and supervision of 8097 out-growers.

Employees and plantations workers are part of the 12,300 inhabitants of PALMCI industrial unit. Schools and dispensaries are free of charge for workers and their families as well as local communities. The presence of palm plantations in this area was a good opportunity to improve transport facilities for the flow of products. Climate is equatorial with four seasons: high dry season (December to February); high rainy season (Mars to July); Small dry season (August to September) and small rainy season (October to November). In the last 10 years, average annual rainfall was around 1500 mm. The vegetation is composed by evergreen forest. The main ethnic group was Akan. Various types of pesticides (described in Additional file 1) were used for crop protection against insects, rodents and fungus.

### Insecticide susceptibility

Larvae of *Anopheles gambiae* s.l. were collected from natural breeding habitats in and around Ehania-V1 between June and July 2013. Larvae were reared to adults for bioassays in Institut Pierre Richet insectarium (IPR) in Bouaké, Central Côte d’Ivoire. All larvae were provided a diet of Tetra Mikromin fish food until adult stage.

WHO-tube bioassays were carried out using impregnated papers of diagnostic concentrations of six insecticides belonging to the four approved classes: one organochlorine (4% DDT), three pyrethroids (0.05% deltamethrin, 0.05% alpha-cypermethrin and 0.75% permethrin), one organophosphate (1% fenitrothion) and one carbamate (0.1% bendiocarb).

Four batches of 25–27 unfed females of wild *An. gambiae* s.l. and the reference laboratory Kisumu strain, which is free of any detectable resistance mechanisms and raised in the same conditions, 2–3 day-old were exposed to insecticide-treated papers for 60 min at 25 ± 2 °C and 80% relative humidity (RH). Papers without insecticide were used as control. The number of mosquitoes knocked down was recorded at 10 min intervals during the exposure period with pyrethroids and DDT, as the knock down rate shown to be a sensitive indicator for early detection of resistance. After the exposure time, mosquitoes were supplied with 10% sugar solution and mortality was scored 24 h later.

### Synergist PBO assays

Complementary synergist assays were performed using piperonyl butoxide (PBO), an inhibitor of monooxygenases, in order to assess the involvement of detoxifying enzymes in the production of resistant phenotypes. Mosquitoes were pre-exposed to 4% PBO-impregnated filter papers for 1 h and then exposed to insecticide-impregnated papers for additional 1 h according to WHO procedures. Final mortalities recorded 24 h later following exposure were compared between the synergized and non-synergized samples. The susceptible Kisumu strain was not synergized. Exposed mosquitoes to insecticides were individually kept on silica gel and stored in refrigerator (−20 °C) for molecular analysis.

### DNA extraction, molecular forms and target site resistance monitoring

Genomic DNA samples from 50 mosquitoes randomly selected among the dead and surviving mosquitoes from WHO tube bioassays were analyzed by polymerase chain reaction (PCR). Genomic DNA was extracted on individual mosquitoes according to Collins et al. [20]. *Anopheles gambiae* s.l. mosquitoes were identified to species according to Scott et al. [21] and Santolamazza et al. [22]. The presence of L1014F and L1014S *kdr* alleles was assessed using allele-specific PCR [23]. The PCR–RFLP diagnostic test was used to detect the presence of the G119S mutation (*ace*-*1* ^*R*^ gene) according to Weill et al. [24].

### Biochemical analysis

Biochemical assays were performed to quantify and assess the amounts of MFO (cytochrome P450) and GSTs, and the activity levels of NSE for both α- and β-naphthyl acetate as described in WHO book [25]. Batches of 30–35 unfed female mosquitoes (2–3 days old) from the Ehania-V1 strain were used per microtitre plate. Each plate also contained 5–10 specimens (2–3 day-old female mosquitoes from the susceptible Kisumu strain for further comparison. These specimens have not been exposed to any insecticides but frozen at −80 °C prior to biochemical analysis.

### Statistical analysis

Mortality rate of each insecticide was calculated as the proportion of dead mosquito 24 h post-exposure at 95% confidence intervals. No correction was performed using Abbott’s formula [26], as the mortality rates in all controls tests were always less than 5%. WHO [2] criteria were used to evaluate susceptible/resistant phenotype of mosquito strains. Thus, mortality rates >98 and <90% indicated susceptibility and resistance, respectively. For mortalities ranged between 90 and 97% resistance is suspected and the presence of resistant genes in the population must be confirmed by further investigation (additional bioassay tests with the same insecticide and/or by conducting molecular or biochemical assays for known resistance mechanisms). Statistical analyses were performed with STATA version 10.4 (Stata Corporation; College Station, TX, USA) and statistical significance was set at 5%.

The time to knock down 50 and 95% (KdT_50_ and KdT_95_) of pyrethroid and DDT-tested mosquitoes were estimated using a log-time probit model with Polo Plus 1.0 software (LeOra Software). The KdT_50_ and KdT_95_ values were also estimated in synergist assays for pyrethroid and DDT. The ratio between KdT_50_ of resistant strains and susceptible strains referred as resistance ratio (RR_50_) was calculated, with Chi square (χ^2^) to compare groups. Mann–Whitney non-parametric test was used to compare the mean enzymatic level/activities of monooxygenases, NSEs and GSTs between the wild *An. gambiae* s.l. from Ehania-V1 and the susceptible Kisumu strain.

## Results

### Mortality and knock-down rates

Mosquitoes displayed variable levels of mortality at 24 h, following exposure to the seven insecticides tested (Table 1). Mortality rates were above 90% for fenithrothion (98.0%) and deltamethrin (97.6%). However, DDT (8.3%), permethrin (39.6%) and alphacypermethrin (63.8%) yielded mortality rate below the WHO-referenced threshold (90%). The mortality rates in the control never exceeded 5% and so there was no need to correct with Abbott’s formula.

**Table 1:** Distribution of mortality to insecticides in *An. gambiae* s.l. 24 h post-exposure

Insecticide	No. tested	No. dead	% dead (95% CI)	Status
Permethrin	101	40	39.6 (30.05–49.15)	R
Deltamethrin	124	121	97.6 (94.88–100)	SR
Alphacypermethrin	105	67	63.8 (54.62–73.00)	R
DDT	108	9	8.3 (3.12–13.54)	R
Fenitrothion	101	99	98.0 (96.10–99.94)	S
Bendiocarb	102	89	87.2 (81.26–93.25)	R

Data in parentheses are 95% confident interval

*No* number, *S* susceptible, *R* resistance, *SR* suspected resistance

The knock-down effects of pyrethroids and DDT on the *An. gambiae* s.l. population from Ehania-V1 compared to susceptible Kisumu strain are summarized in Table 2. The KdT_50_ values for *An. gambiae* Kisumu strain were approximately 12.6, 15.2 and 18.1 min for deltamethrin, alphacypermethrin and permethrin, respectively. KdT_50_ and KdT_95_ values recorded for wild *An. gambiae* s.l. population were approximately 57 and 122.6 min for deltamethrin and 83.9 and 144.7 min for alphacypermethrin, respectively (Table 2). However, KdT_50_ and KdT_95_ could not be determined as less than 50% of mosquitoes tested were knocked down after 60 min.

**Table 2:** Knockdown time (KdT) for 50 and 95% of tested *An. gambiae* Kisumu and *An. gambiae* s.l. Ehania-V1 to pyrethroids and DDT

Insecticide	Mosquito	N	Knockdown time				KdT_50_ ratio	
			kdT_50_ (mn)	CI 95%	kdT_95_ (mn)	CI 95%	RR_50_	CI 95%
Permethrin	Kisumu	104	18.1	16.2–20.3	29.3	24.9–40.4	ND	ND
	Ehania-V1	101	ND	ND	ND	ND		
Deltamethrin	Kisumu	107	12.6	9.2–15.8	27	20.7–48.3	4.55	1.0–12.0
	Ehania-V1	124	57.3	53.7–62.2	122.6	104–155		
Alphacypermethrin	Kisumu	106	15.2	11.2–19.1	34.5	26.1–60.9	5.52	2.5–14.1
	Ehania-V1	105	83.9	72.7–135.9	144.7	104.6–353.1		
DDT	Kisumu	102	48.6	45.9–51.8	99.1	86.7–119.4	ND	ND
	Ehania-V1	108	ND	ND	ND	ND		

*N* sample size, *kdT*_*50*_ knockdown time for 50% mosquitoes, *kdT*_*95*_ knockdown time for 95% mosquitoes, *CI 95%* 95% confidence interval a, *min* minutes, *RR*_*50*_ resistance ratio (kdT_50_ of the tested population/kdT_50_ of the *Kisumu* strain), *R* resistant, *ND* not determined

### Effect of synergist PBO

**Fig. 2:**
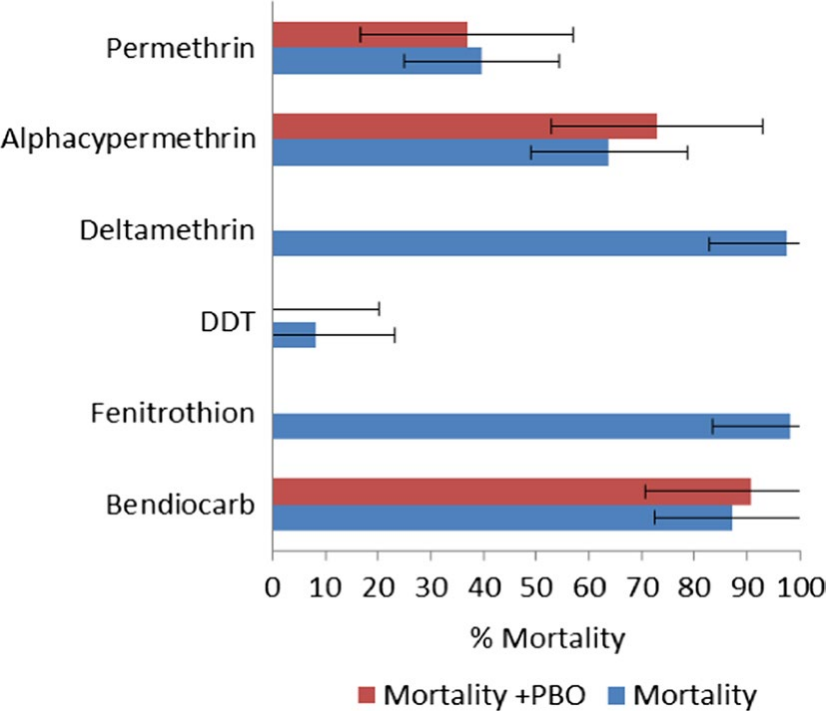
Distribution of mortality rates of *An. gambiae* s.l. with or without PBO in Ehania in 2013

Absence *vs* presence of PBO in insecticide was compared to show the effect of PBO (Fig. 2). There was 3.4 and 9.0% increase in mortality rates recorded with bendiocarb and alphacypermethrin, respectively, when PBO was added, compare to these insecticides alone (bendiocarb: PBO absence = 87.25% vs PBO presence = 90.65% and alphacypermethrin: PBO absence = 63.81% vs PBO presence = 72.81%) without significant difference (p = 0.4325 and p = 0.1629, respectively). However, a slight decrease was observed in permethrin mortality rate, (PBO absence = 39.60% vs PBO presence = 36.89%); (p = 0.69040). Patterning DDT plus PBO a null mortality rate was observed (PBO absence = 8.33% vs PBO presence = 0%); (p = 0. 0032).

### *Anopheles gambiae* species identification and *kdr* L1014F and *ace*-*1*^*R*^ G119S allele frequencies

Of the subset number of *An. gambiae* s.l. mosquitoes (n = 46) analyzed for sibling species identification, only *An. coluzzii* and *An. gambiae* s.s. were found in sympatry in Ehania-V1, and *An. coluzzii* was predominant (72 vs 28%, Table 3). High frequency of *kdr* 1014F mutation was found in both *An. gambiae* (91%) and *An. coluzzii* (96%). The *ace*-*1* G119S mutation was found in these two species populations but at very low frequency (from 12 to 19%) and only at heterozygote state.

**Table 3:** Genotype frequencies of the *kdr* (L1014F) and *ace*-*1* ^*R*^ (G119S) loci in *An. gambiae* s.l. population from Ehania-V1

	*kdr* L1014F				*ace*-*1* ^*R*^ G119S			
	SS	RS	RR	F(1014F)	SS	RS	RR	F(1014F)
*An. gambiae*	0	1	12	0.961	8	5	0	0.192
*An. coluzzii*	0	6	27	0.909	25	8	0	0.121

### Biochemical analyses

Mean level activities of the NSE (α- and β-naphthyl acetate) and mean amounts of MFO (cytochrome P450 s) and GST are presented in Table 4. The mean level activities of NSE for α- and β-naphthyl acetate were 0.081 and 0.084 μmol/mim/mg respectively in Kisumu strain. In wild population from Ehania-V1, mean α- and β-esterase was 0.187 and 0.175 μmol/mim/mg and both values are significantly higher than those recorded in Kisumu strain (p < 0.001). The GST level activity was significantly higher in the wild population from Ehania-V1 than in Kisumu strain (Ehania-V1 = 1.778, Kisumu = 0.226; p < 0.001. The *An. gambiae* s.l. population from Ehania-V1 showed comparable production of MFO (cytochrome P450s) amount to the laboratory susceptible strain Kisumu (Ehania-V1 = 0.119, Kisumu = 0.074; p = 0.277, Ns).

**Table 4:** Mean level of NSE, MFO and GST activity in *An. gambiae* s.l. tested population

	Kisumu		Ehania-V1		*p value*
	N	Mean	N	Mean	
α-Naphthyl acetate (μmol α-naphthol/min/mg protein)	49	0.081 ± 0.032	50	0.187 ± 0.129	<0.001*
β-Naphthyl acetate (μmol β-naphthol/min/mg protein)	45	0.084 ± 0.071	47	0.175 ± 0.120	<0.001*
MFO (nmol P450 unit/mg protein)	47	0.074 ± 0.041	50	0.119 ± 0.113	0.277
GST (μmol GSH conj/min/mg protein)	36	0.226 ± 0.143	44	1.778 ± 1.669	<0.001*

*NSE α* activity with substrate alpha-naphthyl acetate (µmol α-naphthol produced/min/mg protein), *NSE β* level with substrate beta-naphthyl acetate (µmol β-naphthol produced/min/mg protein), *MFO* multiple function oxidase level (nmol equivalent unit of cytochrome P450/mg protein), *GST* GST level (nmol GST conjugated/min/mg protein), *N* number of *An. gambiae* s.l. tested

* Indicated that enzyme activity or quantity was significantly difference compared with the Kisumu reference susceptible strain (p < 0.001)

## Discussion

This study was the first carried out in the area of Ehania-V1 to monitor the susceptibility status of malaria vector *An. gambiae* s.l. to WHO-recommended insecticides. Mortality rates with fenithrothion and deltamethrin was above the threshold set by WHO (90%). While resistance to pyrethroid insecticides is widely reported in Africa and particularly in the majority of the districts of Côte d’Ivoire [18, 27], the results showed apparent susceptibility to deltamethrin at this site in the South Eastern region. More investigation is required to confirm the resistance status of malaria vectors to this insecticide. The highest mortality rate observed with deltamethrin could provide a perspective for control together with organophosphate insecticides in this region. However, the current the results should be treated cautiously for two reasons. First, decrease in resistance to insecticide could result from the declining of selection pressure asserted by the use of this insecticide in a specific area. Indeed, previous report on susceptibility to deltamethrin was made in cotton field area of Korhogo, Northern Côte d’Ivoire following the breakdown of use of this insecticide during the political crisis [28]. This political crisis was also extended to all the country and impacted most agricultural settings of interest. Ehania region was also concerned by this disturbance of regular agricultural practices. Second possible reason could be linked to the frequency of insecticide application within the palm oil farm during the last decade. The study questionnaire administrated to the chief of workers to collect information on pesticides provided a list of insecticides used in Ehania-V1 industrial palm oil farm (Additional file 1). Listed pesticide formulations was also included Decis^®^, a deltamethrin based-product, but its frequency of use remains unknown yet and will also require more clarification in near future. However, a significant increase in knockdown time might be an indicator for the early detection of deltamethrin resistance [8]. Thus, more investigation will be necessary to understand the profile of deltamethrin use in public health and agriculture in the region. The mortality rates to DDT and other pyrethroids such as alphacypermethrin and permethrin were less than 90%. It was impossible to determine this knockdown time especially for permethrin and DDT. However, the knockdown time of wild *An. gambiae* exposed to alphacypermethrin and deltamethrin was more than four times higher compared to the susceptible *An. gambiae* Kisumu strain. In Ehania-V1, *An. gambiae* were also shown to be resistant to bendiocarb but the mortality was seven times lower than the one reported in a previous study from Tiassalé [12].

Similar to other regions of Côte d’Ivoire, including San Pédro, Abidjan, Man, Abengourou, Yamoussoukro and Korhogo [29], both *An. coluzzii* and *An. gambiae* s.s. were found in Ehania-V1 locality within the *An. gambiae* complex with *An. coluzzii* predominant. The abundance of *An. coluzzii* was repeatedly reported in the Southern part of Côte d’Ivoire [12, 15].

Only *kdr* L1014F mutation was detected in *An. gambiae* s.s. and *An. coluzzii* from Ehania-V1, at overall high frequency compared to those reported in the western region of Côte d’Ivoire with low frequencies (0–20%). Findings are similar to studies carried out in the Northern (Korhogo, Kaforo) region with high frequencies of *kdr* L1014F mutation (70 and 80%) [29].

In Tiassalékro (Southern Côte d’Ivoire) where *An. gambiae* showed multiple resistance to insecticide classes, this mutation frequency was high (83%) [12], but below the one observed in Ehania-V1. Furthermore <20% of *ace*-*1* ^*R*^ G119S allele proportion has been found in the study site. The results confirm those of Ahoua et al. [15], which previously showed the absence of homozygous resistant individuals at the *ace*-*1* locus.

Results from synergist assays (PBO) showed no significant effect on mortality rate after pre-exposition to insecticides. Therefore, MFO was probably not involved in resistance in this area in contrast to results from M’Bé, in Central Côte d’Ivoire [30]. Indeed a significant difference in GST and NSE activity was observed when comparing wild strains to the susceptible Kisumu strain. The non-detection of P450 activity in resistance observed in Ehania-V1 appears as a positive outcome for further control strategies as metabolic-based P450 resistance remains a major challenge for the control of malaria vectors in the Africa region.

The strong resistance recorded for DDT, permethrin and alphacypermethrin may be also due to the presence of several environmental pollutants and pesticide residues caused by the extensive use of insecticides to treat palm trees and crops since 1969 [31].

## Conclusion

Resistance of *An. gambiae* s.l. from Ehania-V1 was likely due to *kdr* and probably to esterase activities for alphacypermethrin and *ace*-*1* ^*R*^ for bendiocarb. Multiple mechanisms of resistance in Ehania-V1’s mosquito’s population were suspected and require in-depth investigation. The increase ability of *Anopheles* populations to resist to lethal doses of insecticides has reduced the efficacy of most insecticides used for vector control. Bendiocarb resistance is also emerging in Ehania-V1 mosquitoes and further investigation will be needed to understand the mechanisms. Selection pressure asserted by the use of insecticides in agriculture and probably in public health could be a cause of the resistance in major insecticides in this region. However, an apparent susceptibility to deltamethrin has been identified and if confirm, may precludes perspective for malaria control along with organophosphate in Ehania. These results should be shared with the National Malaria Control Programme in Côte d’Ivoire in order to encourage collaboration with the PALMCI health department toward strong complementary analysis and potential plan for implementation of further control strategies based on deltamethrin and/or organophosphates.

## Additional file

**Additional file 1.** Pesticides use by Integrated farm Unit (IFU) of PALMCI (Ehania-V1). Data were obtained from a questionnaire administered to the responsible of the industrial palm farm.

## Abbreviations

PALMCI: Palm oil Company of Côte d’Ivoire
WHO: World Health Organization
NMCP: National Malaria Control Programme
LLIN: long-lasting insecticide-treated nets
IRS: indoor residual spraying
